# GPU accelerated numerical investigation of the spherical stability of an acoustic cavitation bubble excited by dual-frequency

**DOI:** 10.1016/j.ultsonch.2021.105684

**Published:** 2021-07-27

**Authors:** Kálmán Klapcsik

**Affiliations:** a Budapest University of Technology and Economics, Faculty of Mechanical Engineering, Department of Hydrodynamic Systems, P.O. Box 91, 1521 Budapest, Hungary

**Keywords:** Bubble dynamics, Sonochemistry, Spherical stability, GPU programming

## Abstract

The spherical stability of an acoustic cavitation bubble under dual-frequency excitation is investigated numerically. The radial dynamics is described by the Keller–Miksis equation, which is a second-order ordinary differential equation. The surface dynamics is modelled by a set of linear ordinary differential equation according to Hao and Prosperetti (1999), which takes into account the effect of vorticity by boundary layer approximation. Due to the large amount of investigated parameter combinations, the numerical computations were carried out on graphics processing units. The results showed that for bubble size between $R_{E}=2\;\mu m$ and 4μm, the combination of a low and a high frequency, and the combination of two close but not equal frequencies are important to prevent the bubble losing its shape stability, while reaching the chemical threshold ($R_{\max}/R_{E}=3$) (Kalmár et al., 2020). The phase shift between harmonic components of dual-frequency excitation has no effect on the shape stability.

## 1 Introduction

In a liquid that is irradiated with high-frequency ultrasound, thousands of micron-sized oscillating bubbles are formed. This phenomenon is called acoustic cavitation. Two main categories of acoustic cavitation bubbles exist; namely, the “stable” cavitation bubbles which have relatively long lifetime [3], and the “transient” cavitation bubbles, which tend to disintegrate into daughter bubbles within a few oscillation [4], [5]. These oscillating bubbles show highly nonlinear behaviour [6], [7], [8], [9], [10], [11], [12], [13], [14], [15], [16], [17]. Several studies applied the methods of nonlinear science to investigate this nonlinear behaviour of bubbles [18], [19], [20], [21], [22], [23]. Depending on the frequency and the intensity of the excitation, the bubbles may exhibit various kind of oscillations, e.g., periodic or chaotic. As a result of high Laplace pressure, the oscillation of very small bubbles is suppressed. If the surface tension effects are overcame (Blake’s threshold [24]), the bubble exhibits large expansion. This threshold can be crossed by ultrasound intensity increase or bubble growth. Beyond this threshold bubbles expand many times larger than their equilibrium size in the growth phase, then exhibit violent bubble-collapse due to the inertia of the liquid domain. During the collapse phase, the bubble wall velocity can reach extreme high values and shock waves are generated [25], [26]. Near the minimum radius, the pressure and the temperature inside the bubble can reach 1000bar and 8000K, respectively [27], [19]. This high-energy bubble collapse is usually named as “transient cavitation”. Such high pressure and temperature induce chemical reactions inside the bubble producing various chemical species [28], [29], [30], [31], [32], [2].

A special branch of chemistry, called sonochemistry intends to utilize ultrasound in chemical reactions. For example, one of the keen interests of sonochemistry is the production of $H_{2}$ (green fuel) [33], [34], [35], [36]. Moreover, the produced free radicals via the collapse of bubbles can be used in the degradation and oxidation of pollutants [37], [38], or in wastewater treatment [39], [40], [41], [42], [43]. Other important applications are the hydrolysis of oils [44], [45], the production of nanoalloys or nanoparticles [46], [47], [48], which can be used as catalysts in further reactions.

In contrast with the single frequency excitation, several investigations reported increased chemical yield (even by 300%) by using dual-frequency excitation, due to the synergetic effect of the interacting pressure waves [49], [50], [51], [52], [53], [54]. In the last decades, many theories emerged to explain the influence of multiple excitation frequencies, for example, more active zones are generated in the sonochemical reactor [55], [56] due to the better pattern of the acoustic waves. Others reported the increase of nuclei generated in the bubble cluster [57], [54], increased mass transfer via micromixing [58], the increase of collapse-strength [51], [59], [60], [34] or the lowering of cavitational threshold [61], [62]. The combination and simultaneous resonance properties of dual-frequency driving can also explain the increased collapse strength [63]. The stability properties (e.g., spherical [64] or positional [65] stability) can alter due to the addition of a second driving frequency as well. Although, many studies discuss the beneficial effects, decreased sonochemical output was also reported by using dual-frequency [58]. This indicates that the theoretical understanding of the synergetic effect of dual-frequency is far from complete.

The first steps towards the detailed investigation of the bubble dynamics on a wide range of parameters were made by Hegedűs et al. [66], who published a high-resolution scan in the 6-dimensional parameter space. The total number of parameter combinations was approximately 2 billion. The main control parameters are related to the dual-frequency ultrasonic excitation, these are the two pressure amplitudes and the two frequencies. Secondary parameters are the phase shift between harmonic components and the equilibrium radius of the bubble. The ambient properties; thereby, the material properties were constants. The results showed that the chemical threshold is primarily driven by the bubble size as a parameter. For small (below 3μm), medium (between 3μm and 6μm), and large bubbles (above 6μm), the best choice of frequency combinations are the single frequency with low value (Giant Response [19]), a mixture of low and high frequencies, and a single frequency that is near to the main resonance frequency, respectively. These observations were compared with the results of the present investigations.

Shape stability of the bubbles might play an important role in the optimization of sonochemical reactors since spherical bubbles can produce more focused collapse (e.g., bubble sonoluminescence was boosted 3 times higher [67], [68], [69]. Stronger bubble collapse usually induces higher chemical yield as well [2]. On the other hand, the break-off of a shape unstable bubble enhances the diffusion of the produced chemical species into the liquid domain [70]. Former results [2] also showed that the chemical output is influenced by the bubble size; therefore, the existence of large, shape stable bubbles may be beneficial from the applications point of view. The continuous growth of the bubble is naturally satisfied due to the rectified diffusion [71], [72], [73] of the bubbles, which are oscillating with fairly large amplitude. In this case, the bubbles can grow due to the large diffusive area difference between the expansion and collapse phase. A possible limitation of the bubble growth is the spherical instability. A spherically unstable bubble may undergo different scenarios. It can lose gas by shedding smaller bubbles and regain its shape stability if the fragments disappear. If the fragments are recollected repeatedly, the bubble exhibits “jittering” or “dancing” [74]. Shape unstable bubbles may split into similar sized-fragments or into very small fragments. The latter is called “atomization” observed in bubble trap experiments [75]. Alternatively, shape unstable bubbles may exhibit stable nonspherical oscillations [76], [77].

Shape deformation is induced by a small perturbation that is parametrically excited by the bubble oscillation. This perturbation of bubble shape can be caused by the pressure gradient, the presence of other bubbles or the influence of solid boundaries or liquid surface. Thus, the proper optimization strategies in choosing the parameter combinations require a detailed description of the shape stability properties of the bubbles.

The main aim of the present study is to perform detailed parameter scans in the 6-dimensional parameter space and reveal the shape stable parameter domains. The present formalism follows previous published concept [1], [78]; that is, the surface wave dynamics are described by linear ordinary differential equations which are coupled to a spherical bubble model. Thereby, all mode coefficients behave as linear oscillators driven parametrically via the solution R(t) of the spherical model. Hereby, the radial oscillation is described by the Keller–Miksis bubble model [79], which takes into account the compressibility of the liquid domain that is important in case of high amplitude collapse-like oscillations. Then, the bubble shape distortion is expressed in terms of the sum of spherical harmonic modes [80], [81], [1], [82], [83]. In the present paper, the dynamics of the surface modes were described by decoupled linear ordinary differential equations, which take into account the effect of vorticity generated during bubble collapse by using boundary layer approximation [1]. Based on the good qualitative agreement between linearised models and measurements [81], [76], [84], the oscillation energy transfer between shape and volumetric modes [85], and the interaction between different shape modes, or between the shape distortion and translation motions [86], [85] are neglected. In the present paper, the investigated modes are numbered from 2 up to 6.

For numerical calculations, the MPGOS program package [87] was used and the numerical computations were carried out on 2 NVIDIA GTX Titan Black GPUs. MPGOS is a general-purpose program package written in C++ and CUDA C, and capable to exploit the massive computational power of graphical processing units (GPUs). The numerical results in paper [66] were also obtained by using MPGOS for numerical simulations. It must be emphasized that from the available program packages, at the moment, MPGOS has the best performance for solving a large number of non-stiff, low-dimensional ordinary differential equations on GPU [88], [89]. The capabilities of GPU-accelerated initial value problem solvers has already been demonstrated in papers [90], [91], [92].

It was observed numerically that the chemical output significantly increases above the collapse strength $R_{\max}/R_{E}=3$ [2]. This threshold value is referred as the chemical threshold in the present paper. The obtained numerical results showed two shape stabilizing effect of the dual-frequency excitation for medium-sized bubbles. Spherical bubble collapse above the chemical threshold $R_{\max}/R_{E}=3$ [2] can be achieved by applying the *combination of two close but not equal frequencies*, or the *combination of a low and high frequency driving* signal. The phase shift between harmonic components has no effect on the shape stability.

## 2 Mathematical model

The usually micron-sized bubbles presented in the liquid domain oscillate radially as a result of the ultrasonic irradiation. During the oscillation of small bubbles, due to the effect of surface tension (inversely proportional with the bubble size), the bubbles remain spherical. However, in the case of large bubbles, a small distortion of the bubble surface leads to bubble surface oscillations besides the radial oscillations. In this case, the spherical assumption is not valid anymore; thus, the bubble shape can be described as

(1) $$r(t,\Theta ,\phi )=R(t)+\overset{\infty }{\underset{n=2}{\sum }}a_{n}(t)Y_{n}^{m}(\Theta ,\phi ),$$

where R(t) is the instantaneous mean bubble radius, $Y_{n}^{m}$ is a surface harmonic of degree *n* and order *m*, and $a_{n}$ is the corresponding amplitude of the surface distortion. In the present paper, the investigation is restricted to the exploration of linear stability analysis; thus, the perturbation is independent of the order *m* of spherical harmonics, and decoupled dynamical equations for the surface modes n⩾2 is derived. The interested reader is referred to the papers [93] for more details. The radial oscillation (zeroth mode) is described by the Keller–Miksis equation [79]; and the surface waves are described by ordinary linear differential equations, which takes into account the effect of liquid viscosity by a boundary layer approximation (referred to as BLA model in the present paper) [80], [1] The details of the applied mathematical models are summarized in the following subsections.

### 2.1 Radial dynamics

The Keller–Miksis equation describes the radial dynamics of a bubble in compressible, viscous liquid [79]:

(2) $$\left(1-\frac{\overset{˙}{R}}{c_{L}}\right)R\overset{¨}{R}+\left(1-\frac{\overset{˙}{R}}{3c_{L}}\right)\frac{3}{2}{\overset{˙}{R}}^{2}\;=\left(1+\frac{\overset{˙}{R}}{c_{L}}+\frac{R}{c_{L}}\frac{d}{\mathrm{dt}}\right)\frac{\left(p_{L}-p_{\infty }(t)\right)}{\rho_{L}},$$

where $\rho_{L}=997.1\;\mathrm{kg}/m^{3},c_{L}=1497.3\;m/s$, and $p_{L}$ are the liquid density, the sound speed and the pressure in the liquid at the bubble wall, respectively. The dots stand for derivatives with respect to time. Due to the dual-frequency driving, the pressure far away from the bubble is

(3) $$p_{\infty }(t)=P_{\infty }+P_{A1}\sin(2\pi f_{1}t)+P_{A2}\sin(2\pi f_{2}t+\theta ),$$

where $P_{\infty }=1\;\mathrm{bar}$ is the ambient pressure, $P_{A1}$ and $P_{A2}$ are the pressure amplitudes corresponding to the first and second frequency component; $f_{1}$ and $f_{2}$ are the excitation frequencies and θ is the phase shift.

The pressure inside the bubble is the sum of the partial pressures of non-condensable gas $p_{G}$ and vapour $p_{V}$. The connection between the inner and outer pressures at the bubble wall is described by a mechanical balance of normal stresses written as

(4) $$p_{G}+p_{V}=p_{L}+\frac{2\sigma }{R}+4\mu_{L}\frac{\overset{˙}{R}}{R},$$

where σ=0.072N/m and $\mu_{L}=8.902\cdot 10^{-}4\;\mathrm{Pa}\;s$ are the surface tension, and liquid dynamic viscosity, respectively. The gas pressure is calculated via a polytrophic change of state:

(5) $$p_{G}=\left(\frac{2\sigma }{R_{E}}-p_{V}+P_{\infty }\right){\left(\frac{R_{E}}{R}\right)}^{3\gamma }\text{.}$$

In the above equation, γ=1.4 denotes the polytrophic exponent (adiabatic behaviour), and $R_{E}$ is the equilibrium radius.

### 2.2 Surface waves

For the proper modelling of the surface wave dynamics, a partial differential equation that describes the evolution of the toroid component of vorticity has to be solved [93]. Since the vorticity is considerable only within a boundary layer around the bubble, the integral terms in [93] can be approximated; thus, the numerical difficulties required by a solution of a PDE is omitted. This boundary layer type approximation leads to the following equation for each surface modes

(6) $$\begin{array}{c} {\overset{¨}{a}}_{n}+\left[3\frac{\overset{˙}{R}}{R}-2(n-1)(n+1)(n+2)\frac{\mu_{L}}{\rho_{L}R^{2}}\right)\\ \left(+\frac{2n{\left(n+2\right)}^{2}}{1+2\delta /R}\frac{\mu_{L}}{\rho_{L}R^{2}}\right]{\overset{˙}{a}}_{n}\\ +(n-1)\left[-\frac{\overset{¨}{R}}{R}+(n+1)(n+2)\frac{\sigma }{\rho_{L}R^{3}}\right)\\ \left(+2\frac{\mu_{L}\overset{˙}{R}}{\rho_{L}R^{3}}\left((n+1)(n+2)-\frac{n(n+2)}{1+2\delta /R}\right)\right]a_{n}=0 \end{array}$$

that is referred in the present paper as the BLA model. The comparison of the exact linear formalism and the boundary layer approximation shows a good agreement in the case of small liquid viscosity [94]; thus, it implies that the BLA model is suitable to investigate the linear shape stability. It is worth noting that the omission of the PDE saves a huge amount of computational resources. This is important in case of the exploration of huge parameter space.

According to [82], the boundary layer thickness for large bubbles (R≫δ) is defined as the diffusive length scale ($\sqrt{\mu_{L}/(\rho_{L}\omega )}$) and for small bubbles (R≪δ), the boundary layer thickness can not be larger than the bubble itself; thus, a cut-off is applied as R/2n. In the dual-frequency case, the boundary layer thickness is defined as:

(7) $$\delta =\min\left(\max\left(\sqrt{\frac{\mu_{L}}{\rho_{L}\omega_{1}}},\sqrt{\frac{\mu_{L}}{\rho_{L}\omega_{2}}}\right),\frac{R}{2n}\right),$$

where $\omega_{1}=2\pi f_{1}$ and $\omega_{2}=2\pi f_{2}$ are the angular frequencies.

The above system of equations is transformed into dimensionless form by introducing dimensionless variables; namely, the dimensionless bubble radius $x_{1}=R/R_{E}$; the dimensionless surface wave amplitude $\alpha_{1,n}=a_{n}/R_{E}$; the dimensionless time $\tau =t/(2\pi /\omega_{1})$; the dimensionless bubble wall velocity $x\prime_{1}=x_{2}$ and the dimensionless surface wave velocity $\alpha_{1,n}\prime =\alpha_{2,n}$. The dimensionless system of equations is given in A.

## 3 The numerical procedure

**Fig. 1:**
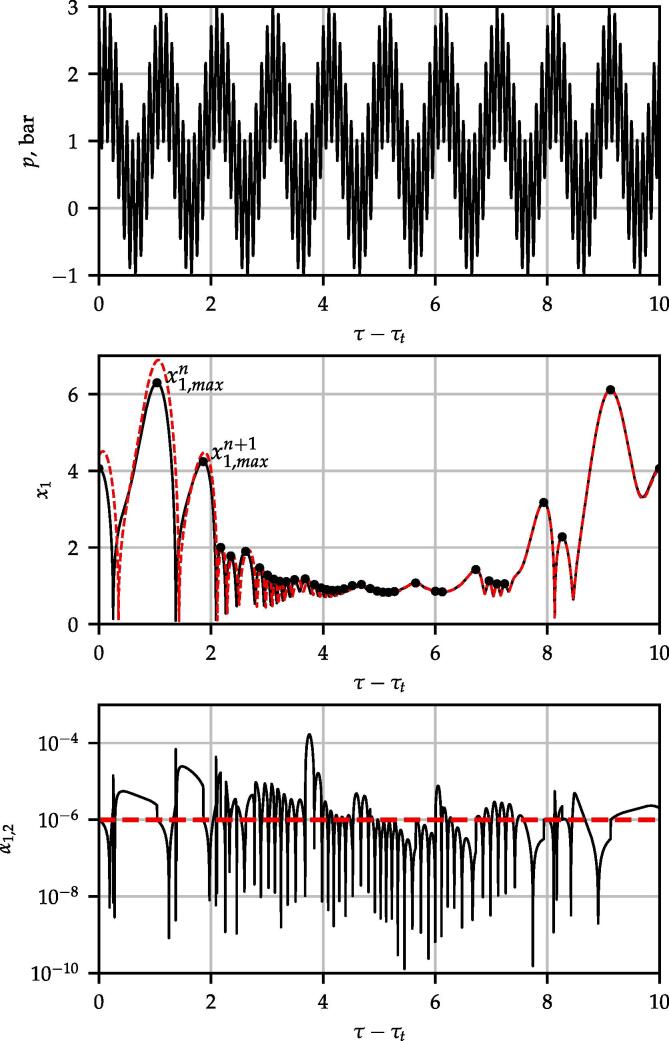
Time series diagrams for parameters $f_{1}=200\;\mathrm{kHz}$ and $f_{2}=20\;\mathrm{kHz}$ frequencies and $P_{1}=P_{2}=1\;\mathrm{bar}$ pressure amplitudes with θ=0 phase shift in case of $R_{E}=3.5\;\mu m$. The top, middle and row panels present the driving signal, the dimensionless bubble radius time curves and the absolute value of dimensionless surface harmonics of mode 2, respectively. The black and red curves correspond to the converged and transient solution, respectively. The black dots represent the consecutive maximum radii, and the vertical red dashed lines denote the value of initial condition for stability analysis.

The numerical procedure follows a similar concept published in [66]. In case of every parameter combinations, initial value problem computations were carried out by using fix initial condition that is $x_{1}(0)=1$ and $x_{2}(0)=0$. An example is given in Fig. 1. The top plane shows the driving signal, and the second shows dimensionless radius time curves. The black curve denotes a converged periodic solution and the red curve shows the transient solution that converges to the black curve. The integration time is measured by the number of bubble oscillations, which means an integration phase between two consecutive local maximum values of $x_{1,\max}^{n}$. The first 1024 oscillations were treated as an initial transient and discarded. It is worth mentioning that the long transient cycles serve only for the numerical purpose to obtain the converged solution of the Keller–Miksis equation. In real cases, the complex dynamics of the bubble cluster does not allow such a long stable oscillation due to bubble–bubble interaction, translational motion or growth via the rectified diffusion. As the surface waves dynamics is described by linear ODEs, which can only describe infinite growth or decay in long term as time goes to infinity, the present results can only state long-term spherical stability of the bubble. Therefore, taking into account the transient phase (1024 collapse) does not affect the overall long-term stability (decay or growth). Consequently, during the transient cycles, the spherical stability of the bubble was not examined. To avoid decay or growth of surfaces modes during the transient phases, the surface distortion amplitude and the corresponding velocity were initialized as $\alpha_{n}(0)=0$ and ${\overset{˙}{\alpha }}_{n}(0)=0$ in the numerical code. The dimensionless time required for the first 1024 collapse is denoted by $\tau_{t}$, and the time series curves in Fig. 1 are shifted by this value. It must be emphasised that the transient solutions may be important for adequate modelling of clusters (bubble–bubble) interaction, and in case of parameter shifting (e.g.,changing the intensity) during the application. However, in this case, the linear models for surface amplitude are insufficient for the proper description of bubble behaviour; thus, nonlinearity (coupling between surface modes and radial oscillation) is not negligible. Such an analysis is beyond the scope of the present study.

As the subject of the present study is the investigation of the linear shape-stability and as the BLA model is based on linear theory, the prescription of the initial conditions for the surface mode perturbations are arbitrary. In the literature, different approaches exist to handle this arbitrariness. For instance, several papers apply fixed initial conditions with prescribed distortion amplitude of $a_{n}(0)=10\;\mathrm{nm}$ or $a_{n}(0)=1\;\mathrm{nm}$ [95], [96], [76], [78]. Another approach is to model the molecular fluctuation by the addition of random or Gaussian distributed noise with prescribed standard deviation during the integration [97], [98] or reformulate the problem as stochastic differential equation [99]. Liu et al. [100] used linear scaling of distortion amplitude with the bubble size as $a_{n}/R_{0}=0.01$. In the present study, a fixed initial condition was applied as follows. As the radius-time curve can exhibit various kind of oscillations (periodic or chaotic due to the nonlinear nature of bubble), instead of integrating over the radial oscillation period to obtain the Floquet multipliers to determine parametric stability [69], [1], the average exponential growth rate was determined. The growth rate is

(8) $$r_{1}=\underset{\tau \to \infty }{\lim}\frac{1}{\tau }\ln\left|\frac{\alpha_{n}(\tau )}{\alpha_{n}(0)}\right|\approx \frac{1}{\tau^{*}}\ln\left|\frac{\alpha_{n}(\tau^{*})}{\alpha_{n}(0)}\right|,$$

where $\tau^{*}$ denotes the dimensionless duration of the 256 consecutive oscillations. Preliminary simulations revealed that above 256 collapses, the average growth rates do not vary with further increase of $\tau^{*}$. Note that the high number of investigated periods are required to obtain the growth rate values accurately. However, letting the integration of the surface dynamics to run up to such a high number of oscillation period, the exponential growth or decay of the surface modes causes numerical problems due to the different range of numbers in the fraction of Eq. (8). Thus, the logarithmic growth of the surface modes was calculated in the following way. At the end of every integration phase, the fraction of the logarithmic growth was calculated and the absolute value of the surface amplitude was set back to the initial condition. The bottom panel of Fig. 1 explains this procedure. The stability analysis begins after the transient iterations at $\tau -\tau_{t}=0$. At this point, a surface perturbation is prescribed with a constant value ($10^{-6}$) denoted by the red horizontal dashed line in the figure. Then, several integration phases are performed. At the end of every integration phase, by exploiting the arbitrariness of the prescription of the initial condition, the distortion amplitude value is prescribed again to $10^{-6}$ and the corresponding distortion velocity was rescaled linearly. The signature of the distortion and the amplitude were not changed. By exploiting the linear nature of this ordinary differential equation, the total logarithmic growth in Eq. (8) equals with the sum of the logarithmic growths corresponding to sub-intervals:

(9) $$\ln\left|\frac{\alpha_{n}(\tau^{*})}{\alpha_{n}(0)}\right|\approx \underset{i}{\sum }\ln\left|\frac{\alpha_{n}(\tau_{i})}{\alpha_{n}(\tau_{i-i})}\right|=R,$$

where the absolute value of $\alpha_{n}(\tau_{i-i})$ equals the initial perturbation; and $\alpha_{n}(\tau_{i})$ denotes the value of the surface mode values at the end of the integration phase, $\tau_{i}-\tau_{i-1}$ denotes the dimensionless duration between integration phases. With this technique, the surface amplitudes are bounded both from below and above, see the bottom panel of Fig. 1. For numerical safety purposes, a specific upper and lower bound was also defined. The upper limit was set $\alpha_{n}=1$ (bubble break-up). The lower bound was $\alpha_{n}=10^{-16}$ to avoid round-off error. If one of the bounds was reached during the integration, a similar normalisation was made. The applied program code is available in the git-hub repository [101]. The compilation of the code requires the MPGOS program package of version 3.1 that can be download from [102].

It is worth mentioning that the growth rate calculated via Eq. (8) depends on the frequency value $f_{1}$, as the dimensionless time depends on $f_{1}$ as well. Therefore, during the evaluation of the numerical results, the frequency dependency of the growth rate values was eliminated. As $\tau^{*}=t^{*}\cdot f_{1}$, from (8), (9), the exponential average growth rate with dimension of 1/s can be written as

(10) $$r=r_{1}\cdot f_{1}=\frac{R}{t^{*}}\text{.}$$

To obtain again a dimensionless quantity, a suitable reference frequency is required to normalise:

(11) $$r_{0}=r_{1}\cdot f_{1}/f_{0},$$

where $f_{0}$ is the linear eigenfrequency of the system [27], [19]:

(12) $$f_{0}=\frac{1}{2\pi }\sqrt{\frac{3\gamma \left(P_{\infty }-p_{V}\right)}{\rho_{L}R_{E}^{2}}-\frac{2\left(3\gamma -1\right)\sigma }{\rho_{L}R_{E}^{3}}}\text{.}$$

### 3.1 The investigated parameter space

In this section, the investigated parameter space is summarized briefly. As the ambient properties (pressure and temperature) were fixed, the material properties were also constant during the simulations. According to the ultrasonic applications, the main control parameters are the frequencies $f_{1},f_{2}$ and pressure amplitudes $P_{A,1},P_{A,2}$ related to the acoustic driving. The frequency values are varied between two orders of magnitude from 20kHz to 2000kHz to resolve the different kinds of resonance properties. The frequency range was divided into 101 values and a logarithmic scale was applied. The pressure amplitude was varied on a linear scale between 0 and 2bar with an increment of 0.1bar. The typical size of the bubbles is a few microns; therefore, it was varied between 1 and 10μm with an increment of 0.5μm. The total number of investigated parameter combinations for a fixed phase shift θ value is (21×21-1)×(101×101)×21≈94.26million. The effect of the phase shift θ is investigated between 0 and 1.75πrad with an increment of 0.25πrad. The ranges of parameters (minimum and maximum values) and the applied resolution with the distribution type (linear or logarithmic) are summarized in Table. 1. The numerical results obtained at $R_{E}=3\;\mu m$ showed that the stability maps are independent of the phase shift (see section 4.2); therefore, the effect of phase shift is investigated only for $R_{E}=3\;\mu m$. Thus, the total amount of investigated parameter combinations is (20+7)×(21×21-1)×(101×101)≈121.19million.

**Table 1:** The investigated parameters space. Ranges, resolutions and types of distribution. The effect of phase shift θ is investigated only for $R_{E}=3\;\mu m$.

	min	max	resolution	scale
$P_{A,1},\;\mathrm{bar}$	0	2	21	linear
$P_{A,2},\;\mathrm{bar}$	0	2	21	linear
$f_{1},\;\mathrm{kHz}$	20	2000	101	logarithmic
$f_{2},\;\mathrm{kHz}$	20	2000	101	logarithmic
$R_{E},\;\mu m$	1	10	21	linear
θ,rad	0	1.75π	7	linear

## 4 Numerical results

**Fig. 2:**
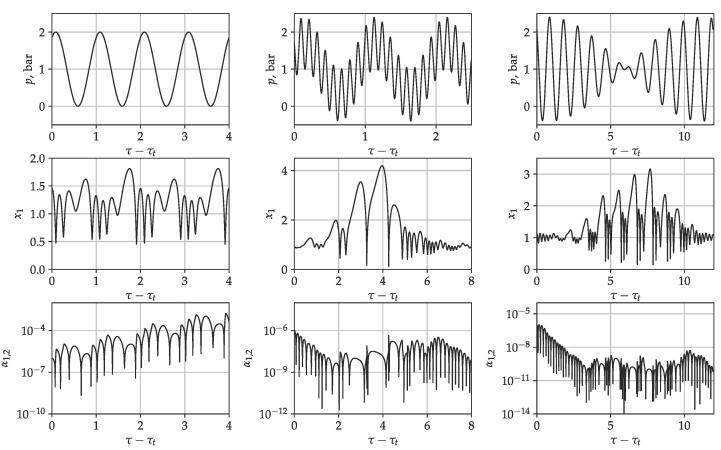
Time series curves for different driving conditions. The first, second and third rows present the driving signal, the dimensionless radius, and the absolute value of surface perturbation as a function of the dimensionless time, respectively.

In Fig. 2, time curves are plotted for bubble size $R_{E}=3\;\mu m$. The first, second and third rows show the driving signal, the dimensionless radius and the dimensionless surface wave amplitude corresponding to the second mode as a function of the dimensionless time, respectively. Note that the dimensionless time is shifted by the $\tau_{t}$ value that denotes the time of the transient iterations. In contrast to Fig. 1, the surface wave amplitude is not normalized in order to get a visual impression of the behaviour of the surface mode. The first column shows the single frequency driving case ($P_{A,1}=1\;\mathrm{bar}$, and $P_{A,2}=0\;\mathrm{bar}$). The driving frequency is $f_{1}=240.45\;\mathrm{kHz}$. The radius time curve shows a simple periodic solution with period 2. Although the collapse strength does not reach the chemical threshold (RE=2 or $x_{1,\max}=3$), the initial perturbation corresponding mode 2 increases over time. In this case, the bubble shape is unstable. Two more cases are plotted in the middle and right column by applying a second driving signal of $f_{2}=27.61\;\mathrm{kHz}$ and $f_{2}=219.3\;\mathrm{kHz}$, respectively. In these cases, the driving amplitudes are $P_{A,1}=P_{A,2}=0.7\;\mathrm{bar}$. It is worth noting that the intensity $I\sim (P_{A,1}^{2}+P_{A,2}^{2})$ in the case of single-frequency and dual-frequency driving is approximately equal. In the dual-frequency cases, the higher peak pressure amplitude in the driving signal induces a larger expansion of the bubbles. Typical responses show a few large-amplitude collapse-like oscillations followed by numerous afterbounces. During the large-amplitude oscillation phase, the perturbation increases. However, during the afterbounces, there is enough time for the decay of the surface perturbation.

Similar computations were carried out on the range of parameters summarized in Table 1 to calculate the average growth rate by means of Eq. (8). As the author believes that the huge amount of numerical data obtained throughout the present study might be useful for further investigations (e.g., calculating diffusive and positional stability domains, then combining the results to obtain complex stability maps such as diagrams of bubble habitat [103], or in case of estimating the chemical output of stable collapses); therefore, the numerical data are published in a public data repository [104]. An accompanying paper discusses the data structure to support their reusability [105]. According to these data, the main observations of the present study are discussed in the following sections.

### 4.1 Data visualization technique

**Fig. 3:**
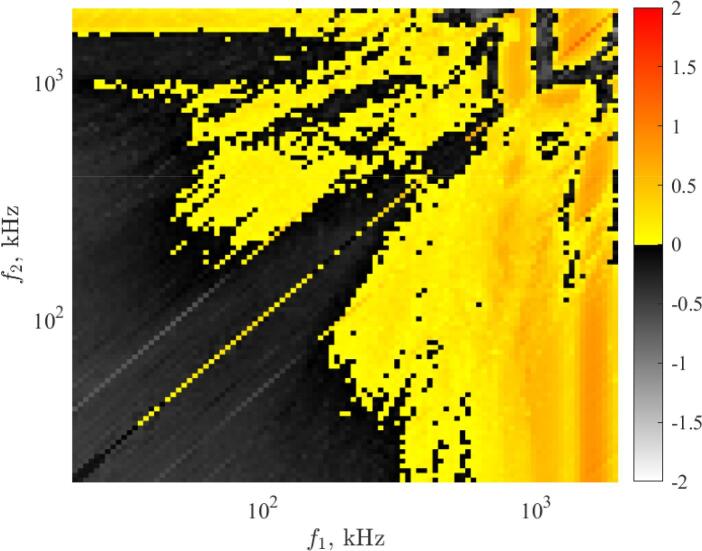
The normalized growth rate corresponding to the second mode as a function of the excitation frequencies at pressure amplitudes $P_{A,1}=1\;\mathrm{bar}$ and $P_{A,2}=0.6\;\mathrm{bar}$ for bubble equilibrium radius $R_{E}=3\;\mu m$ and phase shift θ=0rad.

In the 6-dimensional parameter space, the proper representation of the numerical results is highly challenging. The basic “ingredient” is to create two-dimensional arrays of two-dimensional parameter diagrams for fixed bubble size and phase shift. An example of the construction of such plots is given throughout this section for an equilibrium bubble radius of $R_{E}=3\;\mu m$ and a phase shift of θ=0rad, where the growth rates $r_{0}$ are presented. Figure 3 presents a typical example of a subplot, where the growth rate corresponding to the second harmonic mode as a function of the driving frequencies $f_{1}$ and $f_{2}$ is plotted. The resolution is 101×101, and the scale of the axis is logarithmic in order to properly cower 2 ranges of magnitudes from 20kHz up to 2000kHz. The pressure amplitudes corresponding to the first and second driving frequencies are $P_{A,1}=1\;\mathrm{bar}$ and $P_{A,2}=0.6\;\mathrm{bar}$, respectively. Negative growth rates (grey domain) implies parametric shape stability of the bubble, and the positive growth rate (yellow–red domain) denote shape instability due to parametric growth of surface oscillations. Note that as the phase shift is zero between the harmonic components, the diagonal of equal frequency combinations represents the single frequency driving with pressure amplitude $P_{A}=P_{A,1}+P_{A,2}$.

**Fig. 4:**
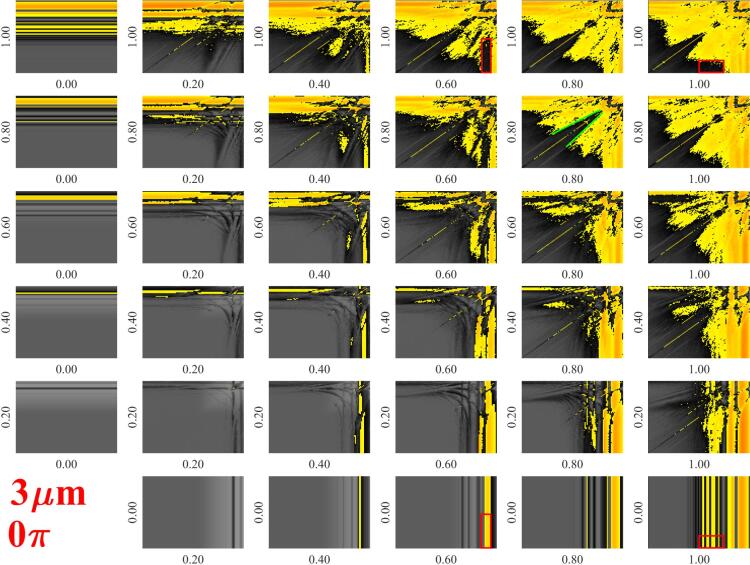
Array of figures of the growth rate subplots corresponding to the second mode. The rows and columns correspond to $P_{A,1}$ and $P_{A,2}$, respectively. Each subplot depicts the growth rate as a function of excitation frequencies $f_{1}$ and $f_{2}$ on logarithmic scale between 20kHz and 2000kHz with a resolution of 101×101.

The parameter diagrams (such as presented in Fig. 3) obtained at different pressure amplitude combinations can be organized into an array of figures. An example with a limited number of pressure amplitude values are plotted in Fig. 4. The columns and rows of the array correspond to $P_{A,1}$ and $P_{A,2}$, respectively. For example, in case of the bottom row, $P_{A,2}=0\;\mathrm{bar}$ (single frequency excitation), while $P_{A,1}$ is increasing from 0.2bar up to 1bar with an increment of 0.2rad. From the bottom row up to the top row, $P_{A,2}$ is increasing in the same manner. The $P_{A,1}=P_{A,2}=0\;\mathrm{bar}$ (equilibrium) is omitted from the array of figures. Note that the array of figures containing pressure amplitude combinations up to 2bar with an increment of 0.2bar are provided in the public data repository [104].

The figure shows that in the case of single-frequency excitation (bottom row) at low $P_{A,1}$ pressure amplitude, the bubble shape is said to be parametric stable since the growth rate decays at every frequency values. By increasing the pressure amplitude up to $p_{A,1}=0.4\;\mathrm{bar}$, near the main resonance (yellow vertical line) the bubble shape becomes unstable, since the second mode exhibit a positive growth rate. Further increasing $P_{A,1}$ leads to a higher domain of unstable regions corresponding to the resonance phenomenon. By adding a second driving component, the unstable shape can be stabilized (see the red rectangles). For example, at $P_{A,1}=0.6\;\mathrm{bar}$ the unstable domain near the resonance becomes stable by adding the second driving component with $P_{A,2}=1\;\mathrm{bar}$ pressure amplitude. Similarly, the unstable domains near the harmonic resonances at $P_{A,1}=1\;\mathrm{bar}$ can be stabilized by adding a second driving component with a small frequency. Another stable domain can be observed (enclosed by the green lines in case of $P_{A,1}=P_{A,2}=0.8\;\mathrm{bar}$) that is two close but not equal frequency excitation. The same effect of dual-frequency driving can be observed at different pressure amplitude combinations as well.

**Fig. 5:**
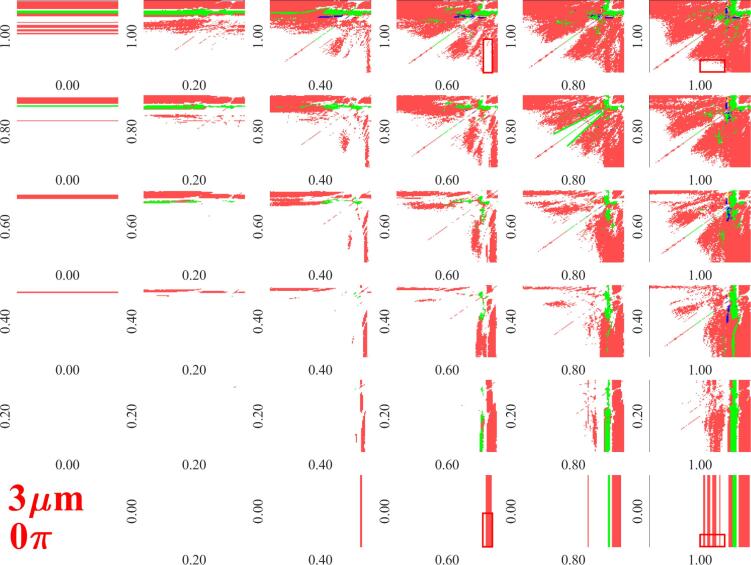
Array of figures of the stability maps. The rows and columns correspond to $P_{A,1}$ and $P_{A,2}$, respectively. Each subplot depicts the most unstable mode as a function of excitation frequencies $f_{1}$ and $f_{2}$ on logarithmic scale between 20kHz and 2000kHz with a resolution of 101×101.

To represent the stability map of the bubbles instead of plotting the growth rate values for each mode separately, the most unstable mode (which exhibits the highest growth rate at a given parameter combination) is plotted as a function of $f_{1}$ and $f_{2}$ frequencies, then the array of figures are composed as demonstrated above. The obtained stability maps are depicted in Fig. 5. The white region denotes the shape stable oscillation (all investigated modes exhibit negative growth rate), the coloured regions denote the unstable modes with the highest growth rate. The red, green and blue colours related to the second, third and fourth harmonic modes, respectively. Note that the fifth and sixth mode is either stable or exhibit lower growth rate than the ones plotted in Fig. 5. Therefore they are not represented here. Additional stability maps for different equilibrium bubble sizes are given as in the data repository [104], where the fifth and sixth modes are colour-coded with yellow and magenta.

The figure shows that at low pressure amplitudes within the investigated frequency range, the bubble shape is stable. However, with increasing pressure amplitude, the size of stable domains gradually decreases. The unstable domains are governed by the second surface mode as known from the literature [69], [78], [96] due to the lower natural frequency. At moderate pressure amplitude, the stabilizing effect of the dual-frequency can be seen. For example, the same shape stable domains can be observed as in case of Fig. 4. The first is the combination of a low frequency with high frequency excitation (an example is highlighted by the red rectangles), the second is the combination of close but not equal frequency (for example, in case of $P_{A,1}=P_{A,2}=0.8\;\mathrm{bar}$ the white region enclosed by green lines.).

### 4.2 Phase shift independence

To investigate the effect of the phase shift, further computations were carried out with the same resolution of frequencies and pressure amplitudes summarized in Table. 1 for equilibrium radius $R_{E}=3\;\mu m$ setup by increasing the phase shift with an increment of 0.25πrad. The stability maps are plotted as an array of figures in a similar way presented in the case of Fig. 5 for every phase shift value. The pressure amplitude values are increasing with an increment of 0.2bar from 0bar up to 2bar. At fixed bubble size, the array of stability maps related to different phase shift values are almost identical except for equal frequencies. In the case of equal frequencies, 0rad or πrad phase shift values result in single frequency excitation with pressure amplitude $P_{A,1}+P_{A,2}$ or zero driving signal, respectively. Therefore, frequency combinations with distinct frequency values are considered in the present study. The figures are concatenated into an animation that is available in the data repository [104] called *Effect*_*Of*_*Theta*_*Animation*_*Re*_*3micron.*gif The independence of collapse strength from the phase shift was also observed in [66]. According to these results, the effect of phase shift is not investigated for different bubble sizes; thus, in the following, the dependence on the bubble size is investigated only with fixed θ=0 phase shift.

**Fig. 6:**
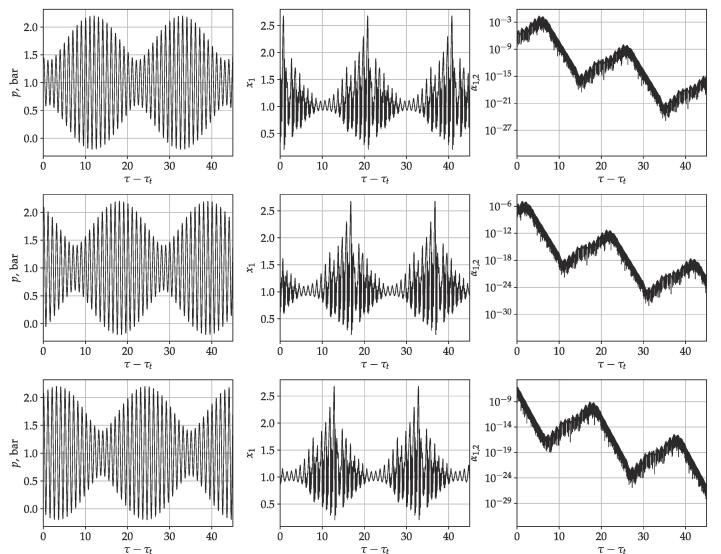
Time series curves for different phase shift values. The first, second and third column depicts the driving signal, the dimensionless radius and the absolute value of surface perturbation as a function of the dimensionless time, respectively. The top, middle and bottom rows correspond to phases shift θ=0,0.5π,and1π, respectively. The parameters are $f_{1}=200\;\mathrm{khz},f_{2}=210\;\mathrm{kHz}$ frequencies, $P_{A,1}=0.8\;\mathrm{bar},P_{A,2}=0.4\;\mathrm{bar}$ pressure amplitude and $R_{E}=3.5\;\mu m$.

Fig. 6 shows three examples of time series curves obtained at different phase shift values to demonstrate the phase shift independence. The graphs in the top, middle and bottom row correspond to phases shifts θ=0,0.5π,and1π, respectively. The driving parameters are $f_{1}=200\;\mathrm{kHz},\;f_{2}=210\;\mathrm{kHz},\;P_{A,1}=0.8\;\mathrm{bar},\;P_{A,2}=0.4\;\mathrm{bar}$. The equilibrium bubble size is $R_{E}=3.5\;\mu m$. The first, second and third column depicts the driving signal, the dimensionless radius and the absolute value of the surface perturbation as a function of the dimensionless time, respectively. Note that the dimensionless time is shifted by $\tau_{t}$ which is the time of the 1024 collapse (transient solution). Aside from the phase shift difference, the driving signals are the same. Since the surface modes are driven parametrically by the solution R(t) of the Keller–Miksis equation and the present model does not take into account the effect of pressure field on the surface wave, the change of the phase shift has no direct effect on the parametric stability of the surface modes. The phase shift independence has already been observed in case of the collapse strength [66].

Although the phase shift has no direct impact on the radial dynamics of the bubble, it has an effect on the sound field in a reaction chamber. Indeed, the pressure field is a composition of the spatial dependent pressure field (transversal or standing wave) and scattered waves of the bubble. Thus, the phase shift may have an impact on the structure formation via the alteration of the pressure field. The detailed investigation of the dependence of the bubble dynamics and the shape stability on the spatial variation of the acoustic field is beyond the scope of the present paper.

### 4.3 Optimal parameter combination for reaching chemical threshold

Further investigation requires a suitable quantity, which measure the strength of the bubble collapses, to identify the chemical threshold. In the literature several approaches exist; for example, the compression ratio $R_{\max}/R_{\min}=x_{1,\max}/x_{1,\min}$ [106], [107], the expansion ratio $R_{\max}/R_{E}=x_{1,\max}$ [108], [109], the quantity of $R_{\max}^{3}/t_{c}$ [51], [59], [110], where $t_{c}$ is the collapse time or the bubble wall Mach number [11]. The present study intends to characterize the magnitude of oscillation by means of the relative expansion

(13) $$\mathrm{RE}=\frac{R_{\max}-R_{E}}{R_{E}}=x_{1,\max}-1\text{.}$$

The numerical investigation of Kalmár et al. [2] revealed that from the above definitions of collapse strength, the relative expansion has the best correlation with the chemical yield. In paper [66], the equivalent pressure amplitude ($P_{A}^{\mathrm{eq}}=\sqrt{P_{A,1}^{2}+P_{A,2}^{2}}$) for reaching relative expansion RE=2 was investigated in the six-dimensional parameter space. The lowest required equivalent pressure amplitude was considered as an optimum parameter setup, as it requires the lowest intensity for reaching chemical threshold. The optimum set of parameters was given as a function of the equilibrium bubble radius $R_{E}$. Hereby, Fig. 4. from the cited paper is replotted; in addition, the growth rate values of the different modes obtained at the optimal parameter setup are also plotted in panel C). The red line in panel A denotes the resonance frequency of the bubble according to Eq. (12), while the red dots in panel B correspond to the lowest required equivalent pressure amplitude $P_{A}^{\mathrm{thr},2}=\sqrt{P_{A,1}^{2}+P_{A,2}^{2}}$ to reach relative expansion RE=2 [75], [66]. This threshold correlates with the minimum threshold of bubble destruction of $R_{\max}/R_{E}=2$ [111] The frequencies $f_{1},f_{2}$ and pressure amplitudes $P_{A,1},P_{A,2}$ corresponding to the optimal setup are denoted by green and blue colours, respectively. In panel C, the growth rate values obtained at the optimal parameter set are plotted as the function of the bubble size. The black, red, green, blue and magenta colours denote the mode number 2, 3, 4, 5 and 6, respectively.

The figure implies that the optimal parameter set for small bubbles (below 3μm) is the giant response with equal frequencies (single frequency excitation). In this range of bubble size, the shape of the bubble is parametrically stable. For large bubbles, the optimal driving frequency is nearly the resonance frequency of the bubble; however, at this size, the bubble shape becomes unstable. This means that long term shape stable oscillation above RE=2 cannot be achieved. Between 3μm and 6μm there is a transition range of the bubble size, where the optimal setup is the mixture of resonance frequency with an additional driving signal with low frequency (giant response). At this moderate range of bubble size, the growth rates are increasing and above $R_{E}=4\;\mu m$ the second mode becomes unstable. Then with increasing bubble size, the modes become gradually unstable, and above $R_{E}=7\;\mu m$ every investigated modes exhibit a positive growth rate in the optimal set of parameters.

### 4.4 Maximum stable collapse

To support ultrasonic applications seeking for strong collapse, the maximum values of the relative expansion for stable bubbles are plotted for different $R_{E}$ values in Fig. 8 as a function of the driving frequencies $f_{1}$ and $f_{2}$. The top left subplot obtained at $R_{E}=1\;\mu m$ and the equilibrium size increases with an increment of 0.5μm up to 5.5μm (bottom right figure). In the yellow–red domain, the relative expansion is above the RE=2 threshold, while in the greyscale domains the collapse strength does not reach the acoustic cavitation threshold. As the investigation is restricted to acoustic driving with distinct frequencies, the equal frequency combination is omitted from the figures, see the diagonal white line. Below 2.5μm (first to rows) stable collapse highly above the threshold can be achieved. The highest collapse strength exists in the giant response region (low-frequency domain).

**Fig. 7:**
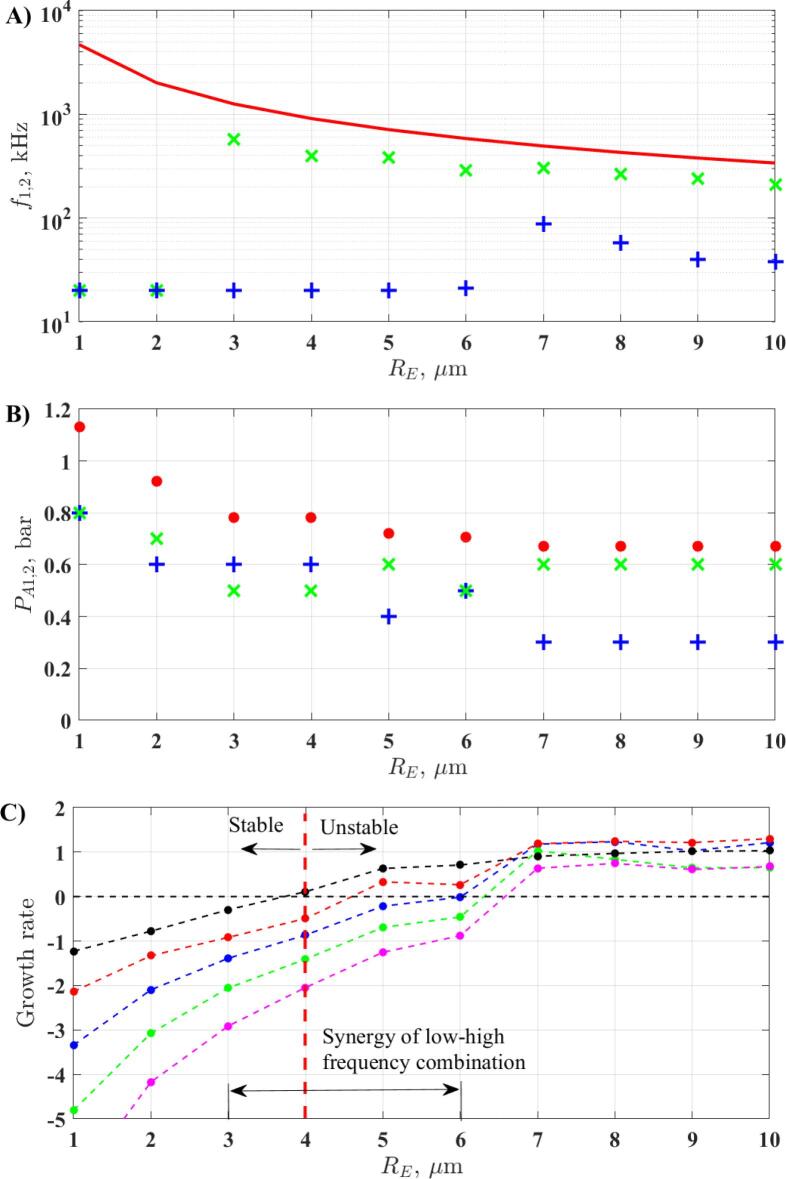
The optimal parameter setup replotted from [66]. Panel A) and panel B) depict the values of the driving frequencies and the pressure amplitudes for reaching chemical threshold RE=2, respectively. The first and second driving components are denoted by the blue and green crosses, respectively. The red line in panel A) is the size-dependent linear resonance frequency of the bubble. The red dots in panel B) represent the lowest values of $P_{A}^{\mathrm{thr},2}$ for reaching chemical threshold RE=2. Panel C) shows the obtained growth rate values at the optimal driving parameters. The black, red, blue, green, and magenta colours correspond to mode number 2, 3, 4, 5 and .6, respectively.

At moderate bubble size, 3-3.5μm (third row), the synergetic effect of dual-frequency can be observed. The mixture of the low and high-frequency driving and close (but not equal) frequency driving induce the maximal stable collapse strength. These observations are in good agreement with the array of stability maps obtained in case of $R_{E}=3\;\mu m$ in Fig. 4, Fig. 5. Although, the strongest collapse can be obtained in the low-frequency region; Fig. 7 shows that for this range of bubble sizes, by combining a low and high-frequency driving, bubbles reach the chemical threshold with the least acoustic intensity. Experimental studies also reported synergetic effect by using similar frequency combinations [68]. For example, the low–high frequency (fundamental and tenth harmonic) combination boosted the sonoluminescence emission by a factor of 2.7. Above this range of bubble size, the domain of mixed-frequency driving narrows. This implies that the optimal set of parameters tend to shift back to the giant response in Fig. 7 for large bubbles as well. For example, in the case of the bubble size $R_{E}=5.5\;\mu m$, stable collapse above the acoustic cavitation threshold is available in the giant response. The figure also shows that the absolute maximum collapse strength in every case is the giant response.

**Fig. 8:**
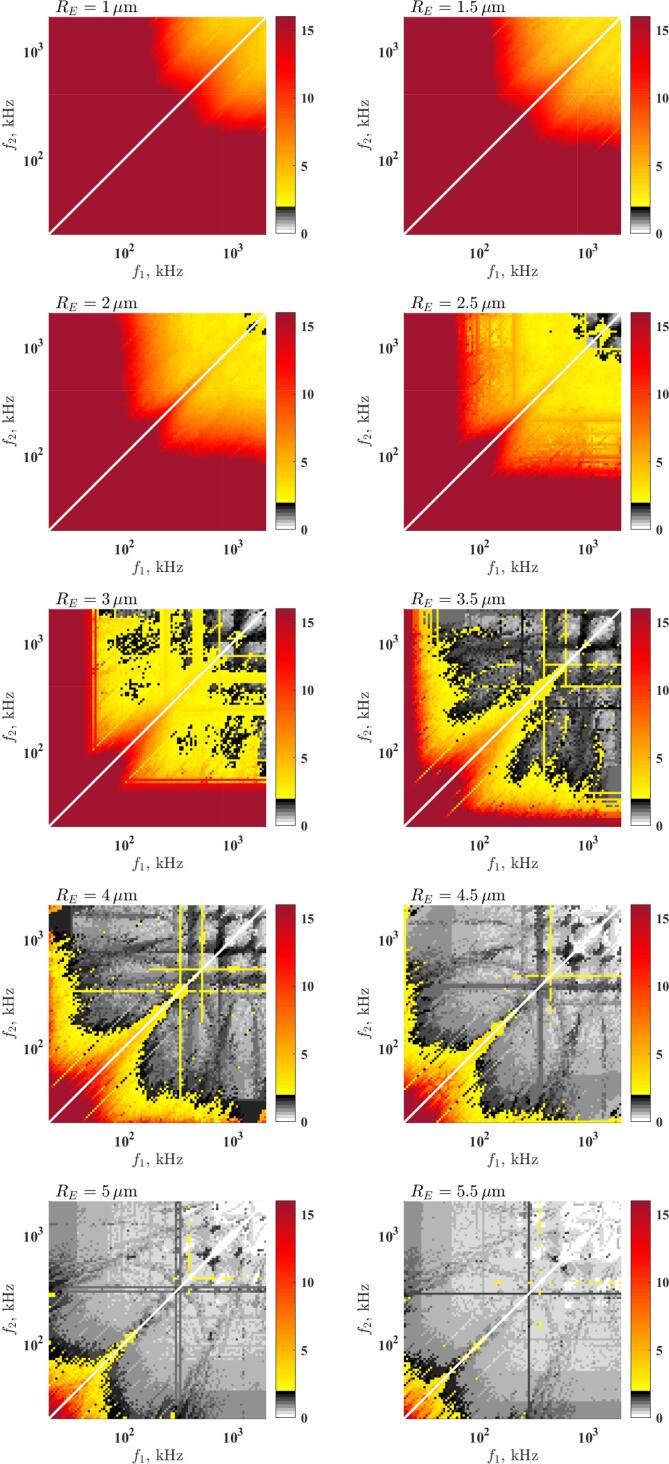
Maximum available stable collapse strength *RE* at different equilibrium bubble sizes $R_{E}$. The greyscale and the yellow–red domains denote the collapse strength below the threshold RE<2, and above the threshold RE>2, respectively.

## 5 Summary and discussion

The shape instability of acoustic cavitation bubbles is a natural limitation of ultrasonic applications. Losing the spherical stability may result in less focused collapse, hereby less chemical activity as well. Moreover, the shape unstable bubbles may break off and disintegrate into smaller bubbles, which are more difficult to excite and their chemical output is less compared to larger bubbles [2]. In the present paper, the shape stability threshold of acoustic bubbles excited by dual-frequency is investigated numerically in a wide range of excitation parameters. The radial oscillation was described by the Keller–Miksis equation and the surface dynamics were described with linear differential equations referred to as the BLA model, which takes into account the vorticity by using a boundary layer approximation [1]. The results of the paper confirm the observations related to the synergetic effect of dual-frequency driving for bubbles below the size of $R_{E}=4\;\mu m$ equilibrium radius. Comparing the single-frequency and dual-frequency cases, the bubble collapse can be stronger in the dual-frequency cases. For example, the sonoluminescence of bubbles can be boosted up to three times higher than the single frequency case [67]. According to the numerical results, the *mixture of two close but not equal frequencies* and the *combination of a low and high-frequency driving* is beneficial for middle-sized bubbles to increase collapse strength, while maintaining the spherical shape. The phase shift between harmonic components has no effect on the shape stability.

The growth rate corresponding to different modes at optimal parameter sets published in [66] showed that for small bubbles the optimal set for reaching the chemical threshold is still the single frequency driving. The synergetic effect of dual-frequency excitation is significant between $R_{E}=3\;\mu m$ and 6μm. Between $R_{E}=4\;\mu m$ and 7μm, the surface modes successively become unstable. This transition in terms of the bubble size raises up two important issues. First, the results in the present paper based on a simple linear model to determine the linear stability of the investigated modes, thus the long-term, stable surface oscillations cannot be captured. However, it is possible to excite the bubble in such a way as to exhibit stable nonspherical oscillations after reaching the linear stability limit due to the effect of nonlinearity [112], [113], [114], [85]. In this case, stable surface oscillations are presented with amplitude smaller than the bubble size itself. Therefore, nonspherical stable bubbles in this range of bubble size can exist; however, instead of focused spherical oscillations, they could exhibit stable nonspherical oscillations. To capture this kind of oscillations, a more complex model, which takes into account the mutual coupling between surface modes and radial dynamics, is necessary. The experimental results in [115] have shown that besides the collapse strength, the mixing of the produced chemical species is required to increase efficiency. During the spherical collapse, the interchange of the chemical species through the bubble wall is mainly governed by diffusion, which is a slow process. Nonspehrical oscillations increase the mixing effect, although the collapse is less focused. Thus, the question is which mode can be excited above the linear threshold to maximize the mixing effect, but still prevent the disintegration of the bubble. The detailed discussion of this issue is beyond the scope of the present paper.

The second issue is related to the bubble break-off due to the surface instability. It is known from the theory of rectified diffusion [71], [72] that the bubbles grow due to the large diffusive area difference between the collapse and growth phase of oscillation that referred to as rectified diffusion. In this sense, bubbles oscillating with high amplitudes will reach the limit of their growth and disintegrate into smaller bubbles (lifetime of acoustic cavitation bubble [116], [117]). A possible strategy to optimize the overall sonochemical efficiency of the reactor is to vary the driving parameters during the operation in order to manipulate the lifecycle of single bubbles or clusters. For example, varying intensity to avoid spherical instability and maintain spherical bubble to achieve more intense collapse, or force the bubble to break off to increase mixing and the number of bubbles in the cluster. In this sense, the shape unstable bubbles behave as a source of daughter bubbles as well and affect the overall behaviour of bubble cluster [118], [119], [120]. It is worth mentioning that besides thermal and radial damping [121], [122] the increase of viscous damping via the application of viscous liquid may be a possible technique to stabilize bubble shape.

The numerical results showed that the maximal collapse strength with spherical bubble collapse can always be achieved in the giant response (low-low frequency). It must be emphasized that in the giant response region, as a result of large positive bubble wall acceleration, small disturbances may be amplified rapidly on the timescale of the collapse. These type of instabilities are classified as *Rayleigh–Taylor* (RT) or *afterbounce* (AB) instability depending on the timescale (main collapse or afterbounce) [82]. However, the arbitrariness in the prescription of the initial condition for surface dynamics would lead to an inexact RT/AB threshold above an uncertain collapse strength value. In addition, the present numerical technique (integrating from max to max) does not distinguish main collapses and afterbounces; therefore, these type of instabilities were not examined. Stability maps corresponding to RT/AB threshold obtained in [94] showed that RT/AB thresholds advance side by side the iso-lines of relative expansions. Another limitation of the extremely high relative expansion is when the solution exceeds Mach number $\mathrm{Ma}=\overset{˙}{R}/c_{L}=1$. Based on the fundamental theory of fluid dynamics, Yasui et al. [123] proved that the Mach number can never exceed unity.

Further limitations of the present model have to be discussed. The mass transfer and the thermal effects are neglected in the present model. The types of mass transfer across the bubble wall are the non-equilibrium evaporation and condensation of vapour and the diffusion of non-condensable gases through the bubble interface [124]. The evaporation and condensation is a two-step process, consist of the phase change at the bubble wall and the diffusion of vapour inside the bubble. The evaporation and condensation take place due to the change of the internal pressure and temperature during the oscillation of the bubble. The high pressure and temperature gradient inside the bubble drive the relative mass diffusion besides the diffusion driven by concentration gradients resulting in inhomogeneous bubble interior [125]. Across the bubble surface, gas diffuses into the bubble during the bubble expansion phase, while at the collapse phase the gas diffuses out of the bubble when the internal pressure is higher than the ambient pressure. In the case of high-amplitude collapse-like oscillations, the inward diffusion overwhelms the outward diffusion; thus, the bubbles continuously grow via the rectified diffusion [71], [72], [73]. This phenomenon may be significant during long cycles. The present investigation neglects the effect of rectified diffusion.

In addition, bubbles in the acoustic field move in the liquid domain due to the Bjerknes forces [126], [127], [86]. The primary Bjerknes force arises due to the pressure gradient of the acoustic fields. The adjacent bubbles interact via their emitted pressure as well. The emitted sound waves inducing a pressure gradient that causes acoustic force analogously to the primary Bjerknes force. It is called the secondary Bjerknes force, which is important in case of close bubbles. Due to the finite speed of sound, a time delay of scattered pressure has to be considered [128], [129]. In the present paper, the such multi-bubble phenomena were not taken into account.

As a final remark, striving for the highest collapse strength is not always a necessary objective. For instance, there is an optimal range of internal temperature between 4000K and 6000K for air bubbles in water, where the production of oxidants is maximal [130], [131]. In a simple configuration of oxygen bubble placed in water, the number of chemical species and the number of chemical reactions are 9, and 44, respectively [2]. The different reactions initiated at different threshold temperature; thus, the composition of the chemical output is different for different collapse strength (temperature). In this sense, the required collapse strength depends on the application. Keep in mind that the correct treatment of the internal temperature [132], [133] and the chemical kinetics [130], [40] inside the bubble or the CFD modelling of bubble collapse [134] requires orders of magnitude higher computational resources.

## CRediT authorship contribution statement

**Kálmán Klapcsik:** Conceptualization, Data curation, Formal analysis, Funding acquisition, Project administration, Investigation, Methodology, Software, Supervision, Validation, Visualization, Writing - original draft, Writing - review & editing.

## Declaration of Competing Interest

The authors declare that they have no known competing financial interests or personal relationships that could have appeared to influence the work reported in this paper.

## Appendix A Dimensionless system of equations

The mathematical model introduced in Section 2 is transformed into a dimensionless form by introducing dimensionless variables. These are the dimensionless bubble radius $x_{1}=R/R_{E}$; the dimensionless surface wave amplitude $\alpha_{1,n}=a_{n}/R_{E}$; the dimensionless time $\tau =t/(2\pi /\omega_{1})$; the dimensionless bubble wall velocity $x\prime_{1}=x_{2}$ and the dimensionless surface wave velocity $\alpha_{1,n}\prime =\alpha_{2,n}$. The dimensionless system is written as

(A.1) $$x_{1}\prime =x_{2},$$

(A.2) $$x_{2}\prime =\frac{N}{D},$$

(A.3) $$\alpha_{1,n}\prime =\alpha_{2,n},$$

(A.4) $$\alpha_{2,n}\prime =-A_{n}\alpha_{1,n}-B_{n}\alpha_{2,n},$$

where $N,D,A_{n},B_{n}$ are

(A.5) $$N=\left(C_{0}+C_{1}x_{2}\right){\left(\frac{1}{x_{1}}\right)}^{C_{10}}-C_{2}\left(1+C_{9}x_{2}\right)-C_{3}\frac{1}{x_{1}}-C_{4}\frac{x_{2}}{x_{1}}-\left(1-C_{9}\frac{x_{2}}{3}\right)\frac{3}{2}x_{2}^{2}-\left(C_{5}\sin(2\pi \tau )+C_{6}\sin(2\pi C_{11}\tau +C_{12})\right)\left(1+C_{9}x_{2}\right)x_{1}\left(C_{7}\cos(2\pi \tau )+C_{8}\cos(2\pi C_{11}\tau +C_{12})\right),$$

(A.6) $$D=x_{1}-C_{9}x_{1}x_{2}+C_{4}C_{9},$$

(A.7) $$A_{n}=-S_{1,n}\frac{x_{2}\prime }{x_{1}}+S_{4,n}\frac{C_{3}}{2x_{1}^{3}}+\frac{C_{4}}{2}\left(S_{4,n}-\frac{S_{3,n}}{1+\frac{\delta }{x_{1}C_{14}}}\right)\frac{x_{2}}{x_{1}^{3}}$$

(A.8) $$B_{n}=\frac{3x_{2}}{x_{1}}+\frac{C_{4}}{2x_{1}^{2}}\left(\frac{S_{2,n}}{1+\frac{\delta }{x_{1}C_{14}}}-S_{4,n}\right)\text{.}$$

respectively. The boundary layer thickness is

(A.9) $$\delta_{n}=\min\left(\max\left(C_{15},C_{16}\right),\frac{x_{1}\cdot C_{14}}{2S_{0,n}}\right)\text{.}$$

The constant, pre-computed coefficients, which are independent of mode number *n*, reads as:

(A.10) $$C_{0}=\frac{1}{\rho_{L}}\left(P_{\infty }-p_{V}+\frac{2\sigma }{R_{E}}\right){\left(\frac{2\pi }{R_{E}\omega_{1}}\right)}^{2},$$

(A.11) $$C_{1}=\frac{1-3\gamma }{\rho_{L}c_{L}}\left(P_{\infty }-p_{V}+\frac{2\sigma }{R_{E}}\right)\frac{2\pi }{R_{E}\omega_{1}},$$

(A.12) $$C_{2}=\frac{P_{\infty }-p_{V}}{\rho_{L}}{\left(\frac{2\pi }{R_{E}\omega_{1}}\right)}^{2},$$

(A.13) $$C_{3}=\frac{2\sigma }{\rho_{L}R_{E}}{\left(\frac{2\pi }{R_{E}\omega_{1}}\right)}^{2},$$

(A.14) $$C_{4}=\frac{4\mu_{L}}{\rho_{L}R_{E}^{2}}\frac{2\pi }{\omega_{1}},$$

(A.15) $$C_{5}=\frac{P_{A1}}{\rho_{L}}{\left(\frac{2\pi }{R_{E}\omega_{1}}\right)}^{2},$$

(A.16) $$C_{6}=\frac{P_{A2}}{\rho_{L}}{\left(\frac{2\pi }{R_{E}\omega_{1}}\right)}^{2},$$

(A.17) $$C_{7}=\frac{R_{E}\omega_{1}P_{A1}}{\rho_{L}c_{L}}{\left(\frac{2\pi }{R_{E}\omega_{1}}\right)}^{2},$$

(A.18) $$C_{8}=\frac{R_{E}\omega_{2}P_{A2}}{\rho_{L}c_{L}}{\left(\frac{2\pi }{R_{E}\omega_{1}}\right)}^{2},$$

(A.19) $$C_{9}=\frac{R_{E}\omega_{1}}{2\pi c_{L}},$$

(A.20) $$C_{10}=3\gamma ,$$

(A.21) $$C_{11}=\omega_{1}/\omega_{2}$$

(A.22) $$C_{12}=\theta ,$$

(A.23) $$C_{13}=2\pi /\omega_{1},$$

(A.24) $$C_{14}=R_{E},$$

(A.25) $$C_{15}=\sqrt{\frac{\mu_{L}}{\rho_{L}\omega_{1}}}\;\mathrm{if}P_{A1}\;\neq \;0,\;\mathrm{else}\;C_{15}=0,$$

(A.26) $$C_{16}=\sqrt{\frac{\mu_{L}}{\rho_{L}\omega_{2}}}\;\mathrm{if}P_{A2}\;\neq \;0,\;\mathrm{else}\;C_{16}=0\text{.}$$

Note that if any of the pressure amplitude is zero $P_{A,i}=0$ (single-frequency excitation), the corresponding constant is zero as well; thus, the above definition of boundary layer thickness (see Eq. (A.9)) simplifies to the single-frequency case. Additional parameters, which depend on *n* are

(A.27) $$S_{0,n}=n,$$

(A.28) $$S_{1,n}=n-1,$$

(A.29) $$S_{2,n}=n\cdot {\left(n+2\right)}^{2},$$

(A.30) $$S_{3,n}=n\cdot (n-1)\cdot (n+2),$$

(A.31) $$S_{4,n}=(n-1)\cdot (n+1)\cdot (n+2)\text{.}$$

The distinction of the above constant parameters is beneficial for increasing the computational efficiency and optimizing the required storage. Each of the concurrent problem has an own set of constants labelled with letter *C*, which are calculated before the execution of the kernel; therefore, the number of required floating point operations at the Runge–Kutta stages are highly reduced. This kind of parameters are referred as “Control Parameters” in the MPGOS program package. Parameters labelled with *S* are also precomputed data; but, the same data value is used by every concurrent threads; thus, instead of storing these values for every parallel tasks as constants (the required storage would be the number of data times the number of threads for each investigated mode), these are stored in the shared memory of GPU that is available for each concurrent threads. In addition, reaching data from shared memory of GPU is usually faster than loading data from the device memory. This type of parameters is called “SharedParameters” in MPGOS. For more details about the application of shared and control parameters, see the accompanying data in brief article [105] or the manual of MPGOS [135]. At a given parameter combination, five modes from mode number 2 up to mode number 6 were investigated. Thus the vector of unknowns is $x^{T}=\left[x_{1},x_{2},\alpha_{1,2}\ldots ,\alpha_{1,6},\alpha_{2,2}\ldots ,\alpha_{2,6}\right]$.
